# RNA Sequence Analysis of Human Huntington Disease Brain Reveals an Extensive Increase in Inflammatory and Developmental Gene Expression

**DOI:** 10.1371/journal.pone.0143563

**Published:** 2015-12-04

**Authors:** Adam Labadorf, Andrew G. Hoss, Valentina Lagomarsino, Jeanne C. Latourelle, Tiffany C. Hadzi, Joli Bregu, Marcy E. MacDonald, James F. Gusella, Jiang-Fan Chen, Schahram Akbarian, Zhiping Weng, Richard H. Myers

**Affiliations:** 1 Department of Neurology, Boston University School of Medicine, Boston, MA, United States of America; 2 Bioinformatics Program, Boston University, Boston, MA, United States of America; 3 Center for Human Genetic Research, Massachusetts General Hospital, Harvard Medical School, Boston, MA, United States of America; 4 Friedman Brain Institute, Department of Psychiatry, Icahn School of Medicine at Mount Sinai, New York, NY, United States of America; 5 Program in Bioinformatics and Integrative Biology, University of Massachusetts Medical School, Worcester, MA, United States of America; 6 Department of Biochemistry and Molecular Pharmacology, University of Massachusetts Medical School, Worcester, MA, United States of America; 7 Genome Science Institute, Boston University School of Medicine, Boston, MA, United States of America; Hokkaido University, JAPAN

## Abstract

Huntington’s Disease (HD) is a devastating neurodegenerative disorder that is caused by an expanded CAG trinucleotide repeat in the Huntingtin (*HTT*) gene. Transcriptional dysregulation in the human HD brain has been documented but is incompletely understood. Here we present a genome-wide analysis of mRNA expression in human prefrontal cortex from 20 HD and 49 neuropathologically normal controls using next generation high-throughput sequencing. Surprisingly, 19% (5,480) of the 28,087 confidently detected genes are differentially expressed (FDR<0.05) and are predominantly up-regulated. A novel hypothesis-free geneset enrichment method that dissects large gene lists into functionally and transcriptionally related groups discovers that the differentially expressed genes are enriched for immune response, neuroinflammation, and developmental genes. Markers for all major brain cell types are observed, suggesting that HD invokes a systemic response in the brain area studied. Unexpectedly, the most strongly differentially expressed genes are a homeotic gene set (represented by Hox and other homeobox genes), that are almost exclusively expressed in HD, a profile not widely implicated in HD pathogenesis. The significance of transcriptional changes of developmental processes in the HD brain is poorly understood and warrants further investigation. The role of inflammation and the significance of non-neuronal involvement in HD pathogenesis suggest anti-inflammatory therapeutics may offer important opportunities in treating HD.

## Introduction

Huntington’s Disease (HD) is a devastating neurodegenerative disorder characterized clinically by involuntary choreic movement, personality changes, and premature death[1,2]. The disease is caused by an expanded CAG repeat in the *Huntingtin* gene (*HTT*)[3] that produces selective neuronal loss in the brain[4]. Individuals commonly present characteristic motor signs in mid-life with a mean onset age of 40 years[5]. No therapy to date has definitively delayed onset or subsequent progression of these symptoms. Most studies in HD are conducted using model systems, (i.e. cell lines or mouse models) or peripheral human biospecimens such as blood and not in involved brain regions from human HD affected individuals. While collecting and analyzing human post-mortem samples presents challenges, the study of brain regions involved in HD provides relevant insight into the disease pathogenesis.

Although transcriptional dysregulation has been convincingly implicated in HD[6,7], few genome-wide gene expression studies have targeted affected tissues in post mortem human brain to date. To expand our understanding of alterations in mRNA transcriptomics, we have performed mRNA expression profiling by next-generation sequencing in human post-mortem prefrontal cortex Brodmann area 9 (BA9) in 20 HD and 49 neuropathologically normal individuals using Illumina high-throughput sequencing (See Tables 1 and 2). Although the primarily affected brain region in HD is the striatum[4], neuronal loss of up to 90% by the time of death impedes the interpretation of expression profiles derived from striatal whole tissue homogenate since the cell type distribution is altered from that of corresponding unaffected control tissue. It is well established that the prefrontal cortex is involved in HD pathogenesis[8,9] but suffers substantially less neuronal death than striatum[10]. The brains used in this study have been comprehensively characterized for pathological involvement through detailed histological examination as previously described[11], which enables direct interpretation of the results in the physiological context of neurodegeneration. We therefore used whole tissue homogenate from the BA9 region in this study.

**Table 1:** HD sample statistics.

Sample ID	PMI	Age of Death	RIN	mRNA-Seq reads	Age of Onset	Duration	CAG	Vonsattel Grade	H-V Striatal Score	H-V Cortical Score
H_0001	37.25	55	7.1	7,46,35,390	44	11	45	3	2.661	0.922
H_0002	5.75	69	7.5	7,10,15,288	63	6	41	3	2.644	1.081
H_0003	20.5	71	7.0	7,73,85,918	52	19	43	3	2.428	1.707
H_0005	19.15	48	6.9	8,23,66,794	25	23	48	4	3.820	1.939
H_0006	**unk**	40	6.2	7,71,23,676	34	6	51	4	3.522	1.431
H_0007	8	72	8.5	6,32,94,390	55	17	41	3	2.593	0.849
H_0008	21.3	43	7.4	7,10,56,116	28	15	49	3	2.701	1.701
H_0009	3.73	68	7.8	6,61,69,262	45	23	42	3	2.668	1.701
H_0010	6.16	59	8.3	6,53,41,820	35	24	46	3	2.621	1.200
H_0012	12.75	68	6.0	8,31,10,358	52	16	42	3	2.661	1.077
H_0013	25.1	57	6.1	7,13,20,688	40	17	49	3	2.911	1.491
H_0539	14.5	54	6.5	12,42,22,130	42	12	45	3	2.132	0.401
H_0657	24.3	61	8.1	13,67,64,622	36	25	45	4	3.290	1.604
H_0658	11	48	7.8	8,55,91,704	42	6	44	3	2.410	0.978
H_0681	19.06	69	7.0	7,84,93,314	50	19	42	3	2.484	1.088
H_0695	16.15	55	7.9	8,64,12,654	36	19	45	4	3.581	2.062
H_0700	15.66	50	8.0	7,83,29,378	33	17	47	3	2.741	1.202
H_0726	14.75	50	9.2	8,60,25,890	27	23	48	4	3.598	1.201
H_0740	13.58	75	6.4	10,19,97,434	60	15	42	3	2.621	2.361
H_0750	16.16	53	6.0	12,29,09,122	38	15	48	4	3.260	1.010

**Table 2:** Control sample statistics.

Sample ID	PMI	Age of Death	RIN	mRNA-Seq reads
C_0012	19	66	7.1	11,83,27,116
C_0013	15	69	7.8	8,94,78,160
C_0014	21	79	8.0	6,53,77,604
C_0015	10	61	8.2	12,37,46,070
C_0016	20	58	8.4	6,77,58,208
C_0017	21	70	8.2	7,22,38,818
C_0018	17	66	8.5	6,46,88,322
C_0020	24	60	7.9	8,36,96,384
C_0021	26	76	7.3	7,94,87,172
C_0022	17	61	7.8	7,31,33,936
C_0023	18	62	6.6	9,44,93,436
C_0024	26	69	8.7	6,29,89,822
C_0025	25	61	8.1	5,58,10,684
C_0026	11	88	7.1	7,25,81,752
C_0029	13	93	6.4	5,93,86,108
C_0031	24	53	7.3	7,32,83,170
C_0032	24	57	8.3	7,09,94,352
C_0033	15	43	7.5	6,95,05,712
C_0034	14	71	7.8	6,59,79,612
C_0035	21	46	7.6	6,23,00,754
C_0036	17	40	7.5	6,39,61,372
C_0037	28	44	8.3	6,02,88,132
C_0038	20	57	7.7	6,10,19,098
C_0039	15	80	7.3	7,48,92,650
C_0050	2	74	8.5	8,53,10,070
C_0053	2	69	8.4	16,70,44,880
C_0060	2	76	7.5	10,39,52,680
C_0061	3	78	7.6	9,53,93,100
C_0062	2	87	8.7	8,37,73,400
C_0065	2	86	8.7	11,57,14,502
C_0069	24	54	8.3	12,84,59,102
C_0070	19	68	6.3	14,50,87,692
C_0071	21	106	7.6	8,68,40,836
C_0075	23	52	7.4	9,99,46,984
C_0076	30	46	8.2	8,58,90,116
C_0077	21	36	8.5	8,01,03,722
C_0081	26	55	7.6	8,29,17,984
C_0082	18	57	7.8	12,31,18,398
C_0083	32	66	8.4	8,06,96,360
C_0087	19	64	8.7	7,71,98,978
C_0002	2	73	7.7	12,01,08,434
C_0003	2	91	7.9	3,84,20,004
C_0004	2	82	8.6	7,58,50,406
C_0005	2	97	9.1	15,06,61,916
C_0006	5	86	8.6	6,36,07,838
C_0008	2	91	8.7	6,61,31,458
C_0009	3	81	6.0	6,92,84,092
C_0010	2	79	8.4	6,05,42,776
C_0011	2	63	6.5	9,37,02,684

Statistical analysis of the dataset yielded a large set of 5,480 differentially expressed (DE) genes, which prompted us to develop a novel hypothesis-free geneset enrichment method to categorize the large gene lists into functionally and transcriptionally relevant groups. Our computational analytic approach, using Gene Ontology, biological pathway database, and transcription factor regulatory gene sets, implicates groups of related genes and functions that expose and visually organize the fundamental molecular dysfunctions of the disease. Our computational analytic approach implicates a complex profile of genes related to development, most notably HOX genes, strongly reinforces a fundamental role for neuroinflammation in the HD brain, and expands our understanding of cellular involvement in the disease to implicate all major brain cell type as opposed to one of primarily neuronal degeneration.

## Results

### Widespread Differential Expression Changes Are Observed in HD

**Fig 1:**
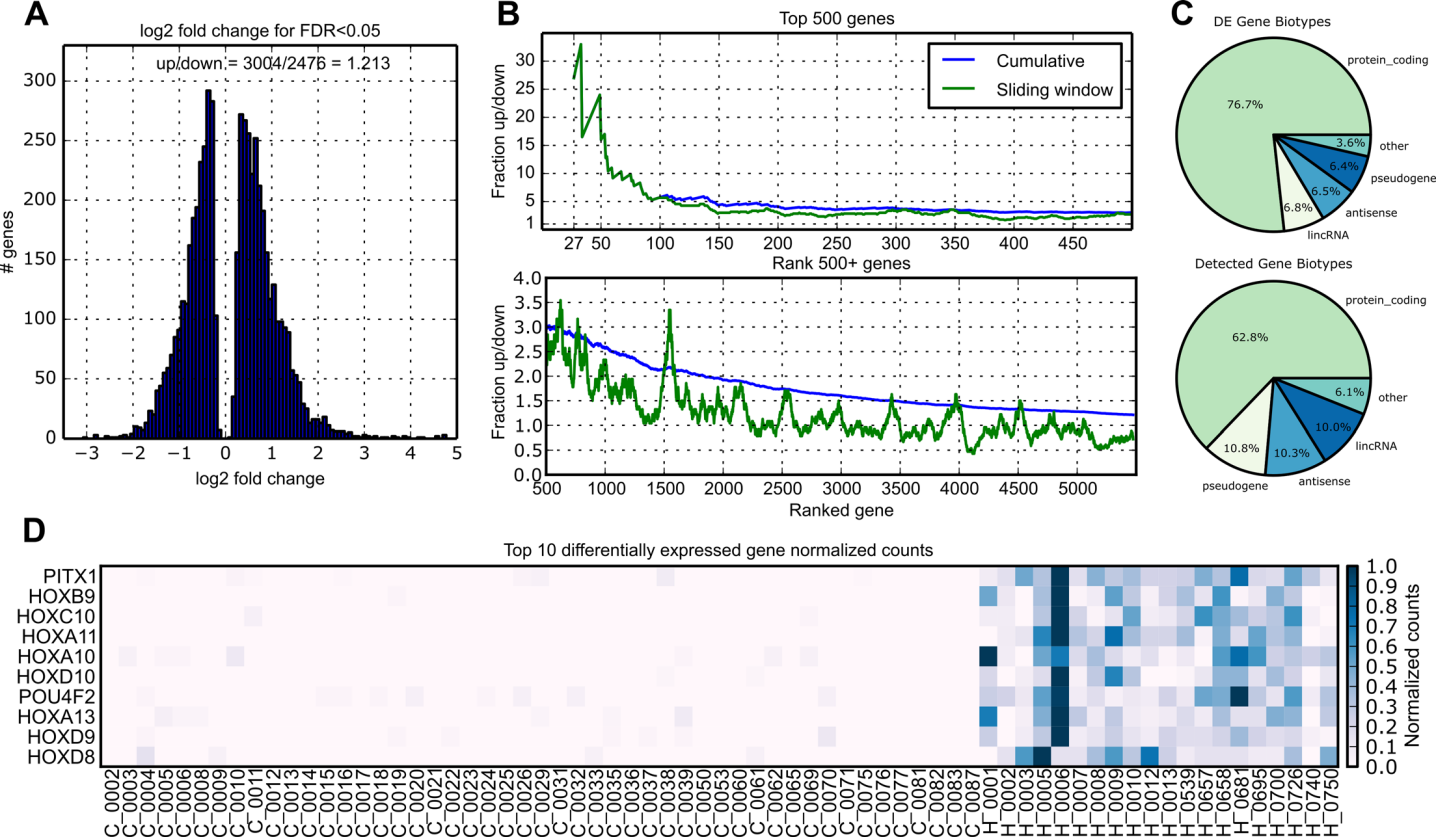
DE statistics. A) Histogram of log2 fold changes for DE genes showing that 54.8% of the DE genes are overexpressed in HD cases. B) Fraction of up vs down regulated genes across the gene list ranked by significance. Top and bottom plots are top 500 and remaining genes, respectively. Sliding windows lines plot the fraction up vs down in the 100 gene window of greater rank than the x coordinate. This plot shows that the most highly differentially expressed genes are predominantly over-expressed in HD relative to control BA9. C) Pie chart shows proportions of biotypes for DE genes according to Ensembl. Protein coding genes are over-represented among the DE genes. D) Normalized counts for all samples in HD and control for top ten significant genes. Rows are normalized for visualization such that the highest count is equal to 1. These genes are almost exclusively expressed in HD cases.

After processing sequencing data to reduce noise, remove outliers, and normalize (see Methods), differential expression (DE) analysis identified 5,480 out of 28,087 confidently expressed genes with significantly altered expression at FDR p-values<0.05 in HD vs control samples, described in Fig 1. More genes are overexpressed in HD versus control than are underexpressed (3,004 vs 2,476, Fig 1A), and this effect is consistent across the whole list of DE genes ranked by significance (Fig 1B). 76.7% of the DE genes are protein coding according to the Gencode v17 annotation[12], while the remaining most abundant biotypes include lincRNAs, pseudogenes, and anti-sense transcripts. A greater portion of DE genes is protein coding when compared to the distribution of biotypes in all 28,087 detectable genes as shown in Fig 1C. Notably, the top DE genes are expressed almost exclusively in HD as illustrated in Fig 1D. A complete list of DE genes is in Table A of S1 File.

With so many DE genes, it is useful to sort the results in such a manner as to expose meaningful sets of relevant genes. As described in Table 3, the top genes sorted by significance are predominantly located in the Hox clusters and other related developmental genes, a novel result also recently observed for HD in our miRNA study[10]. Twenty-four of the 39 *HOX* genes across all four Hox clusters are DE. A table of the Hox genes and their DE properties is included in Table F in S1 File. The majority of these genes are expressed almost exclusively in HD (see Table 3 and Fig 1D), and consequently attain high significance. However, the relative transcript abundance of these genes is low (e.g. *HOXB9* has 8.72 normalized reads on average in the HD samples when the median normalized read count average is 96.6). We sought to identify genes that are both highly expressed and have a large statistically significant difference in expression between HD and control. We created a “differential expression score” (DES) that combines mean expression level, log2 fold change, and statistical significance of differential expression to generate a set of genes that may be relevant to the toxic HD cellular milieu. Table 4 presents the list of the top genes ranked by DES.

**Table 3:** DE genes by significance.

Ensembl ID	Gene Symbol	Overall Mean Counts	HD Mean Counts	Control Mean counts	log2 FC	pvalue	padj	DES
ENSG00000069011.10	PITX1	5.645675	18.68429	0.323793	4.769658	9.57E-39	2.69E-34	903.9895
ENSG00000170689.8	HOXB9	2.542841	8.723281	0.020213	4.76079	1.63E-25	2.29E-21	249.8732
ENSG00000180818.4	HOXC10	2.801117	9.515088	0.060721	4.573976	2.91E-24	2.72E-20	250.6672
ENSG00000005073.5	HOXA11	1.968121	6.790017	0	4.704005	3.92E-24	2.75E-20	181.0905
ENSG00000253293.3	HOXA10	3.490951	11.39924	0.263077	4.273311	8.03E-24	4.51E-20	288.5972
ENSG00000128710.5	HOXD10	2.571228	8.771584	0.040471	4.602451	1.35E-23	6.33E-20	227.1957
ENSG00000151615.3	POU4F2	3.275095	10.65475	0.262991	3.962235	3.42E-23	1.37E-19	244.7754
ENSG00000106031.6	HOXA13	2.456714	8.029653	0.182045	4.165899	6.20E-23	2.18E-19	190.9965
ENSG00000128709.10	HOXD9	2.226869	7.18692	0.202358	3.657288	1.22E-18	3.80E-15	117.4429
ENSG00000175879.7	HOXD8	1.709838	5.601477	0.121413	3.86684	2.09E-18	5.88E-15	94.09001
ENSG00000152779.12	SLC16A12	55.42204	167.6664	9.608012	3.513877	4.74E-18	1.11E-14	2717.727
ENSG00000106004.4	HOXA5	2.198025	7.087533	0.202308	3.879624	4.49E-18	1.11E-14	119.0033
ENSG00000113196.2	HAND1	1.939326	6.244745	0.182012	3.703297	1.46E-17	3.16E-14	96.95744
ENSG00000171540.6	OTP	3.20356	9.16907	0.768658	2.998538	3.93E-17	7.88E-14	125.8704
ENSG00000056736.5	IL17RB	1311.101	2144.334	971.0062	1.392757	3.80E-16	7.12E-13	22182.16
ENSG00000163817.11	SLC6A20	173.0366	433.2822	66.81386	2.355393	2.49E-15	4.37E-12	4629.918
ENSG00000197757.7	HOXC6	1.32181	4.411567	0.060685	3.608891	4.26E-15	7.04E-12	53.19922
ENSG00000183943.5	PRKX	604.7496	900.2916	484.1202	1.419149	6.22E-15	9.20E-12	9471.658
ENSG00000112303.9	VNN2	25.7452	62.90119	10.57949	2.490891	6.03E-15	9.20E-12	707.7395
ENSG00000180229.8	HERC2P3	1987.225	3987.18	1170.917	2.068673	8.09E-15	1.14E-11	44991.92

**Table 4:** DE genes by DES. Differential Expression Score (DES) is calculated as (overall mean counts) x abs(log2 FC) x–log10(adjusted p-value)

Ensembl ID	Gene Symbol	Overall Mean Counts	HD Mean Counts	Control Mean counts	log2 FC	pvalue	padj	DES
ENSG00000197971.10	MBP	180740.9	103940.8	212087.9	-1.14454	0.000227	0.003282	513821.5
ENSG00000131095.7	GFAP	139594.9	147197.9	136491.6	0.747036	0.001561	0.013498	194980.9
ENSG00000120885.15	CLU	98559.44	117016.8	91025.83	0.557853	0.000197	0.00296	139030.9
ENSG00000135821.11	GLUL	61547.89	76676.16	55373.08	0.671273	0.000218	0.003176	103210.9
ENSG00000104833.6	TUBB4A	20856.71	13003.3	24062.19	-0.84178	3.44E-08	4.12E-06	94539.22
ENSG00000171885.9	AQP4	20362.81	27513.91	17443.99	1.094429	2.29E-06	0.0001	89094.63
ENSG00000152661.7	GJA1	13340.95	19835.51	10690.11	1.263084	7.06E-08	6.94E-06	86931.93
ENSG00000168309.12	FAM107A	38970.09	47446.88	35510.18	0.737032	0.000164	0.002585	74321.76
ENSG00000134294.9	SLC38A2	5448.303	9251.666	3895.909	1.312784	3.02E-13	2.83E-10	68291.6
ENSG00000079215.9	SLC1A3	26782.89	35129.11	23376.27	0.855477	6.42E-05	0.001294	66171.29
ENSG00000198668.6	CALM1	83743.27	75824.67	86975.35	-0.34932	0.000542	0.006243	64492.7
ENSG00000160014.12	CALM3	47941.46	38247.79	51898.06	-0.55962	0.000424	0.005225	61221.65
ENSG00000124942.9	AHNAK	9570.149	14157.49	7697.765	1.190373	9.48E-08	8.73E-06	57631.07
ENSG00000226958.1	CTD-2328D6.1	16679.1	5983.11	21044.81	-1.19344	0.000217	0.003174	49731.65
ENSG00000154146.7	NRGN	39663.72	30172.8	43537.57	-0.69835	0.002654	0.019734	47221.27
ENSG00000007237.13	GAS7	15300.17	11322.25	16923.81	-0.69125	5.64E-07	3.50E-05	47122.17
ENSG00000078804.8	TP53INP2	6501.307	3430.574	7754.667	-1.37796	8.45E-08	8.02E-06	45652.02
ENSG00000180229.8	HERC2P3	1987.225	3987.18	1170.917	2.068673	8.09E-15	1.14E-11	44991.92
ENSG00000111674.3	ENO2	25831.65	20005.15	28209.81	-0.57404	6.29E-05	0.001273	42930.44
ENSG00000131711.10	MAP1B	37563.64	29736.15	40758.53	-0.51967	0.00057	0.006441	42770.9

A number of key proinflammatory genes appear as DE in this dataset. Four of the five NFkB family members *NFkB1* (log2 fold change 0.32, q = 0.004), *NFkB2* (LFC 0.73, q = 0.001), *RELA* (LFC 0.63, q = 5.6e-5), and *RELB* (LFC -0.56, q = 0.005) are DE in this dataset. When we examine the 20 interleukin-related genes in the DE gene list, we find that fifteen are cytokine receptors (including *IL17RB*, *IL13RA1*, *IL4R*). However, the cytokines that correspond to these receptors are not DE, nor are *TNFalpha* or *IL6*, two primary cytokines of the immune and inflammatory response.

An independent set of 33 HD and 31 control prefrontal cortex brain samples not used in the sequencing study were subjected to Reverse transcriptase quantitative PCR (RT-qPCR) to replicate the findings of two genes found to be DE in this study. *HOXC10* and *NFKBIA*, genes associated with developmental and neuroinflammatory processes, respectively, were chosen for the replication. *HOXC10* mRNA species were not detected in any of the control samples, whereas 11 HD samples showed amplified product after 40 PCR cycles (p = 0.0002). The presence of *HOXC10* mRNA transcripts in HD, and absence in controls, is consistent with the sequencing findings. In the 16 HD and 16 control samples selected for highest mRNA quality, *NFKBIA* was detected in all samples and, after filtering outlier replicates, was found to be significantly more abundant in HD samples (T = -1.804, p = 0.041).

RT-qPCR was used to quantify and orthogonally validate mRNA differential expression from sequencing. Six genes were selected for the study *AHNAK*, *AQP4*, *SLC38A7C*, *GJA1*, *TP53INP2* which had high DES scores, and *PITX1*, which was the most significantly differentially expressed gene. 21 controls and 15 HD samples from the sequencing study were selected for the assay. Four of the six genes were statistically significant (*AHNAK* p = 0.02; *SLC38A7C* p = 0.01, *TP53INP2* p = 0.03, *PITX1* p = 3.4e-10). Two genes did not meet significance (*AQP4* p = 0.08, *GJA1* p = 0.08). All differential expression was in the expected direction.

### Immune Response, Development, and Transcriptional Regulation Functions Are Enriched in HD

**Fig 2:**
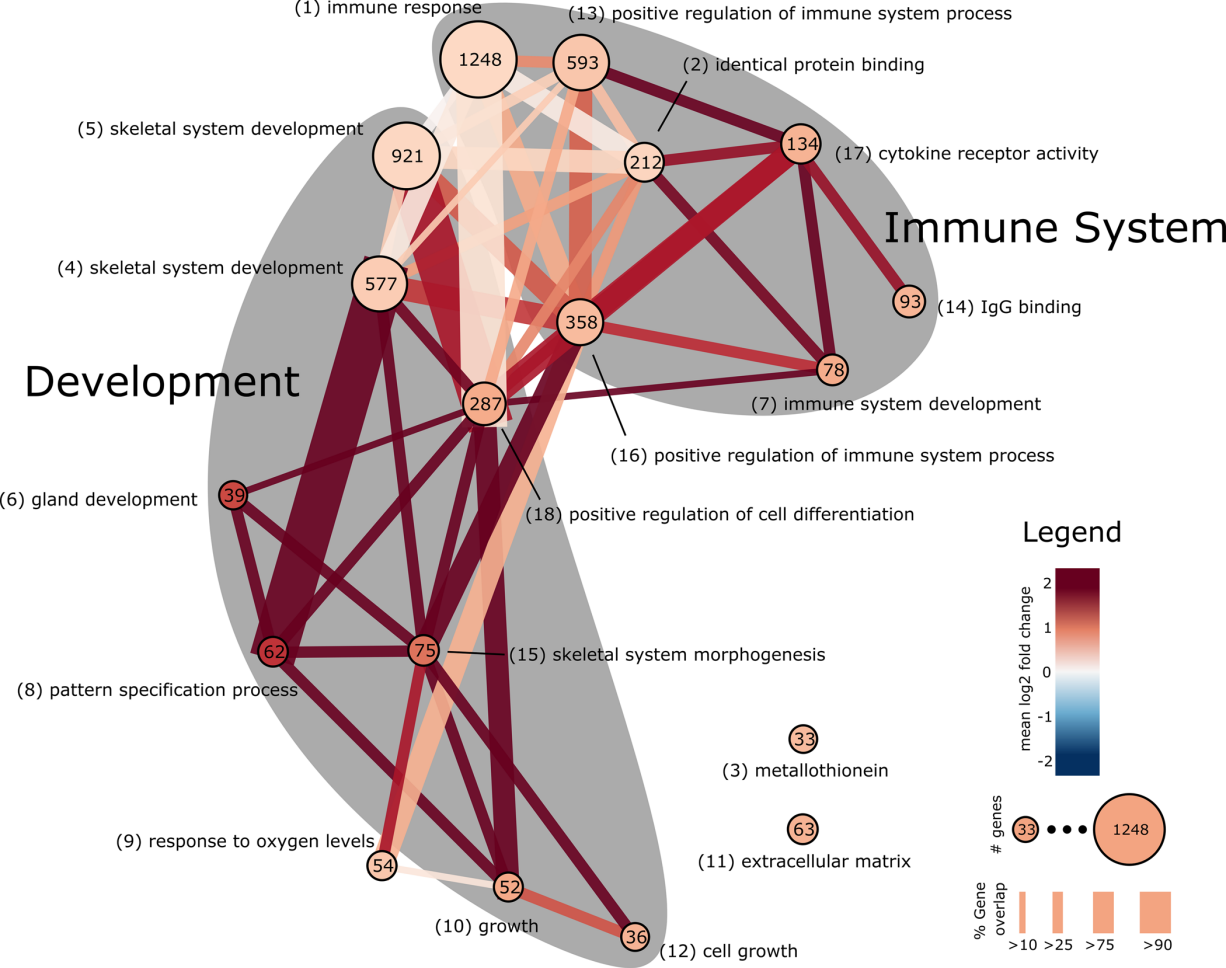
DAVID functional clustering network. Network representation of the DAVID clusters from Table 5. Nodes represent clusters, the size of the node is proportional to the number of unique genes that make up the cluster and numbers within nodes are the number of unique genes mapped to terms in the cluster. Edges between nodes indicate the existence of overlapping genes, where the width is proportional to the percent overlap of genes in the smaller of two connected nodes. The color of nodes and edges is proportional to the average fold change of the genes in the node or edge.

We sought to explore which biological processes are enriched among DE genes in HD. These analyses were performed using the DE list of 5,480 genes ranked by significance. DAVID Functional Enrichment Clustering[13,14] of the top 3000 DE genes (*the DAVID tool restricts the input list size to 3000 genes) identifies numerous biological functions related to immune response, development, cell growth, and transcriptional regulation. Table 5 contains a summary of the enriched clusters identified by DAVID that are significant at a cluster score corresponding to FDR p<0.05. DAVID does not enforce mutually exclusive gene membership between GO categories/pathways and thus one finds redundancy in the list of clusters. The themes of immune response, development, and transcriptional regulation are seen as the most consistent functional groups in this analysis. Fig 2 depicts the functional clusters identified by DAVID as a network where nodes are the DE genes underlying the clusters and edges represent common genes between clusters. The cluster with the largest number of genes is immune response with 1,248, followed by skeletal system development with 921.

**Table 5:** DAVID functional clustering. Cluster Function labels were assigned manually by inspecting the terms within the cluster but generally correspond to the name of the most enriched term within the cluster. The (1) Immune response cluster contained 27 distinct terms from across the default genesets used by DAVID.

#	**Cluster Function**	**Cluster Term Keywords**	**# genes**	**# terms**	**score**
1	immune response	membrane, plasma, transmembrane, receptor	1248	27	3.764689
2	identical protein binding	protein, activity, identical, function	212	5	3.346027
3	metallothioniens	metal, binding, ion-binding, cluster	33	17	3.338415
4	skeletal system development	morphogenesis, embryonic, regulation, development	577	80	3.186388
5	skeletal system development	regulation, transcription, process, negative	921	76	3.143774
6	gland development	development, gland, mammary, lactation	39	3	2.793014
7	immune system development	myeloid, differentiation, leukocyte, cell	78	11	2.637665
8	pattern specification process	symmetry, determination, pattern, left/right	62	5	2.39939
9	response to oxygen levels	response, oxygen, ovulation, process	54	4	2.374104
10	growth	growth, regeneration, developmental, tissue	52	4	2.325598
11	extracellular matrix	extracellular, matrix, proteinaceous, part	63	4	2.27691
12	cell growth	growth, cell, developmental	36	3	2.222128
13	positive regulation of immune system process	response, immune, regulation, activity	593	113	2.204324
14	IgG binding	binding, receptor, c2-type, protein	93	11	2.191733
15	skeletal system morphogenesis	development, morphogenesis, differentiation, bone	75	14	2.170232
16	positive regulation of immune system process	regulation, cell, positive, immune	358	177	2.076509
17	cytokine receptor activity	Fibronectin, type-iii, receptor, regulation	134	32	2.025433
18	positive regulation of cell differentiation	development, morphogenesis, tube, regulation	287	89	2.008837

### Integrated Geneset Enrichment Analysis Identifies Specific Enriched Functional Categories

The DAVID results, while informative, did not provide sufficiently detailed information to understand how the DE gene list mapped to biological functions. To attain a more fine-grained understanding of the enriched biological functions and characteristics of the DE genes, we next performed a detailed analysis of subsets of the DE gene list using the Gene Ontology (GO) annotation database[15] and the MsigDB[16] C2 Canonical Pathways and C3 Transcription Factor target gene sets (see Methods). Briefly, the central idea of the method is to partition the gene list into groups that include increasing numbers of DE genes, where the first group contains the top 25 DE genes, the second group the top 50, and so on for the entire gene list. The last group contains all 5,480 DE genes. Each of these groups is then used to calculate enrichment against each geneset separately using an appropriate statistical method (see below), and then the results from each gene set are concatenated and hierarchically clustered.

### GO Enrichment Analysis Implicates Development and Immune Response

**Fig 3:**
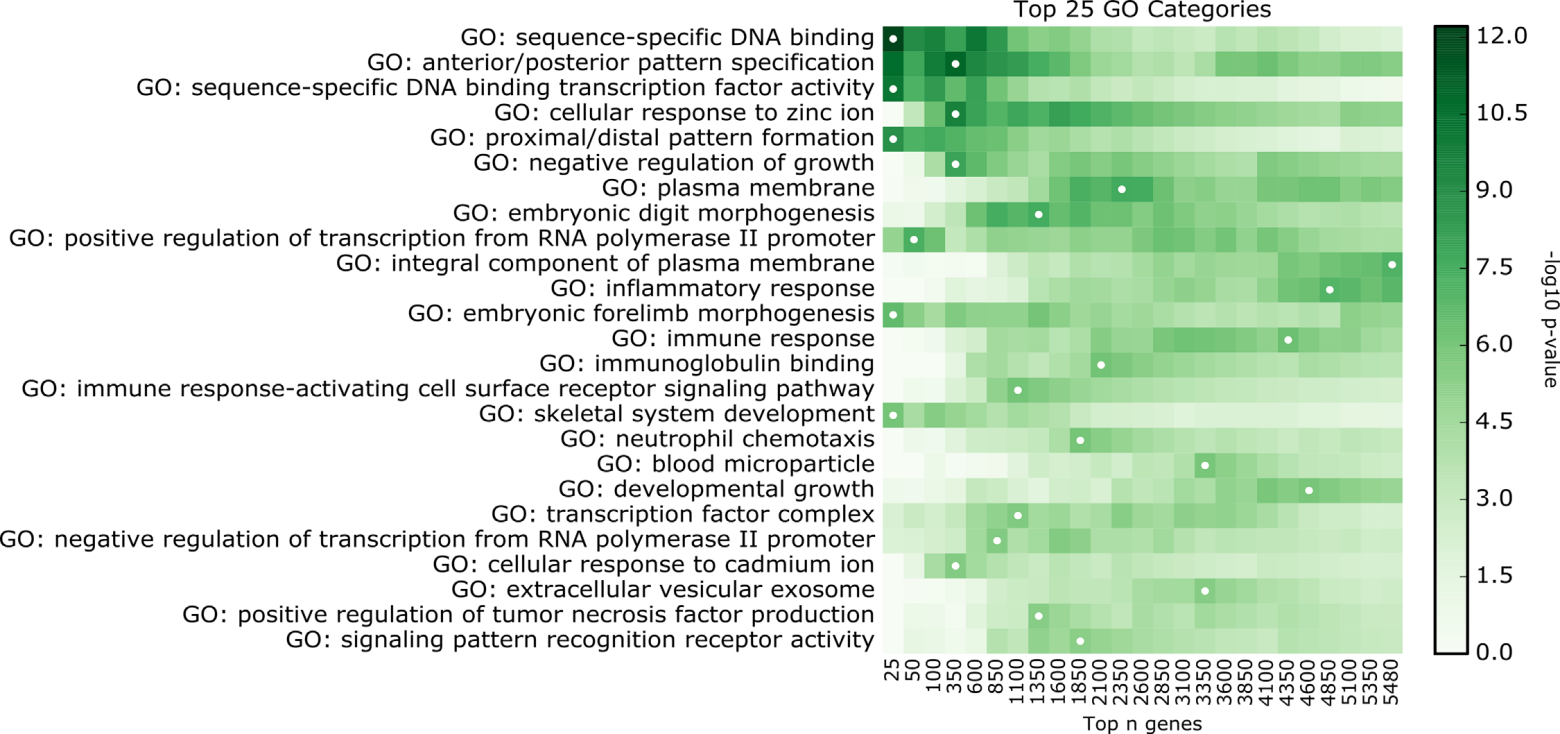
Detailed GO enrichment. Top 25 enriched GO categories across all three GO namespaces identified by topGO for different numbers of DE genes. X-axis indicates the number of top genes used for the enrichment in each GO category, e.g. the first column uses the top 25 genes, the second column uses the top 50, and so on. The intensity is proportional to–log10(p-value) from topGO. White dots indicate the gene set with the most significant p-value, concordant with Table 5. This figure shows that the first three GO Categories are defined by genes that are among the top 25 to 150 DE genes in the dataset. GO Categories further down the list are defined by genes whose differential expression is less pronounced between HD and controls.

GO term enrichment was calculated using topGO[17], a tool that uses the GO term hierarchy to identify enrichment of the most biologically specific categories given a gene list. Fig 3 depicts GO term enrichment of ranked subsets of genes ordered by the most significant term across all subsets. Enrichment is only shown for gene subset/term pairs that attain significance at p<0.05. In total, 901 biological process (BP) terms, 168 molecular function (MF) terms, and 68 cellular component (CC) terms were found to be significant in at least one of the ranked gene subsets. Performing analysis on subsets of top enriched genes reveals that developmental processes and transcriptional regulation are enriched among the most DE genes, while immune response genes are found throughout the DE gene list. Table 6 contains detailed statistics on the top enriched GO terms. These detailed results are consistent with the cluster results from DAVID and better expose the specific biological functions involved in the DE gene list.

**Table 6:** Enriched GO Categories. The most enriched GO category GO:sequence-specific DNA binding using the top 25 DE genes ranked by significance. The second most enriched GO category, GO:anterior/posterior pattern specification, was found when considering the top 350 DE genes.

GO Category	Top n genes	-log10(p-value)
GO: sequence-specific DNA binding	25	12.211851
GO: anterior/posterior pattern specification	350	10.890978
GO: sequence-specific DNA binding transcription factor activity	25	10.19469
GO: cellular response to zinc ion	350	9.630161
GO: proximal/distal pattern formation	25	8.874839
GO: negative regulation of growth	350	7.983699
GO: plasma membrane	2350	7.603115
GO: embryonic digit morphogenesis	1350	7.542254
GO: positive regulation of transcription from RNA polymerase II promoter	50	7.350002
GO: integral component of plasma membrane	5480	7.167156
GO: inflammatory response	4850	7.057813
GO: embryonic forelimb morphogenesis	25	6.633754
GO: immune response	4350	6.311688
GO: immunoglobulin binding	2100	6.178673
GO: immune response-activating cell surface receptor signaling pathway	1100	6.135233
GO: skeletal system development	25	6.059758
GO: neutrophil chemotaxis	1850	6.038185
GO: blood microparticle	3350	5.968453
GO: developmental growth	4600	5.939322
GO: transcription factor complex	1100	5.636701
GO: negative regulation of transcription from RNA polymerase II promoter	850	5.624786
GO: cellular response to cadmium ion	350	5.593366
GO: extracellular vesicular exosome	3350	5.489169
GO: positive regulation of tumor necrosis factor production	1350	5.481418
GO: signaling pattern recognition receptor activity	1850	5.366574

### Pathways Involved in Multiple Immune System Processes Are Enriched

To identify biological pathways as opposed to functional categories, we performed hyper-enrichment of the MsigDB C2 Canonical Pathways using a hypergeometric test on the same ranked subsets of genes as in the GO analysis. These analyses found 538 significantly enriched pathways in at least one gene subset. Enriched Canonical Pathways show a clear immune response and inflammation-related pattern across pathway databases, including Reactome[18,19] innate immune system [DOI: 10.3180/REACT_6802.2], KEGG[20] complement and coagulation cascades [hsa04610] and cytokine-cytokine receptor interaction [hsa04060], and PID[21] IL4-mediated signaling events [Pathway id:il4_2pathway] and NFkB canonical pathways [Pathway id:nfkappabcanonicalpathway].

### DE Genes Are Enriched as Targets of Transcription Factors Implicated In HD

We next performed transcription factor (TF) target analysis using the MsigDB C3 TF regulation gene set to identify potential regulators responsible for the observed differential expression. 237 TFs were identified as significantly enriched in at least one gene subset. A number of the enriched TFs are known to physically interact with the mutant *Htt* protein, including *SP1*[22] and *TBP*[23]. The pattern of enrichment for the top TF, *MYC*-associated zinc finger protein (*MAZ*), tracks closely with pathways associated with immune response (i.e. both become more enriched as more genes are included) but otherwise has no previous connection with HD. The second most enriched TF is forkhead box O4 (*FOXO4*). Another notable enriched TF is *NFkB*, which plays a key role in innate immune response, is critical for glial and neuronal cell function and synaptic signaling[24,25] and impairs synaptic transport in the presence of mutant Htt protein[26]. Other TFs implicated as potential regulators of the DE genes include *NFAT*[27], *HSF1*[28], and *PU1*[29].

### Integrated Geneset Enrichment Analysis Links Biological Function and Transcriptional Regulation

**Fig 4:**
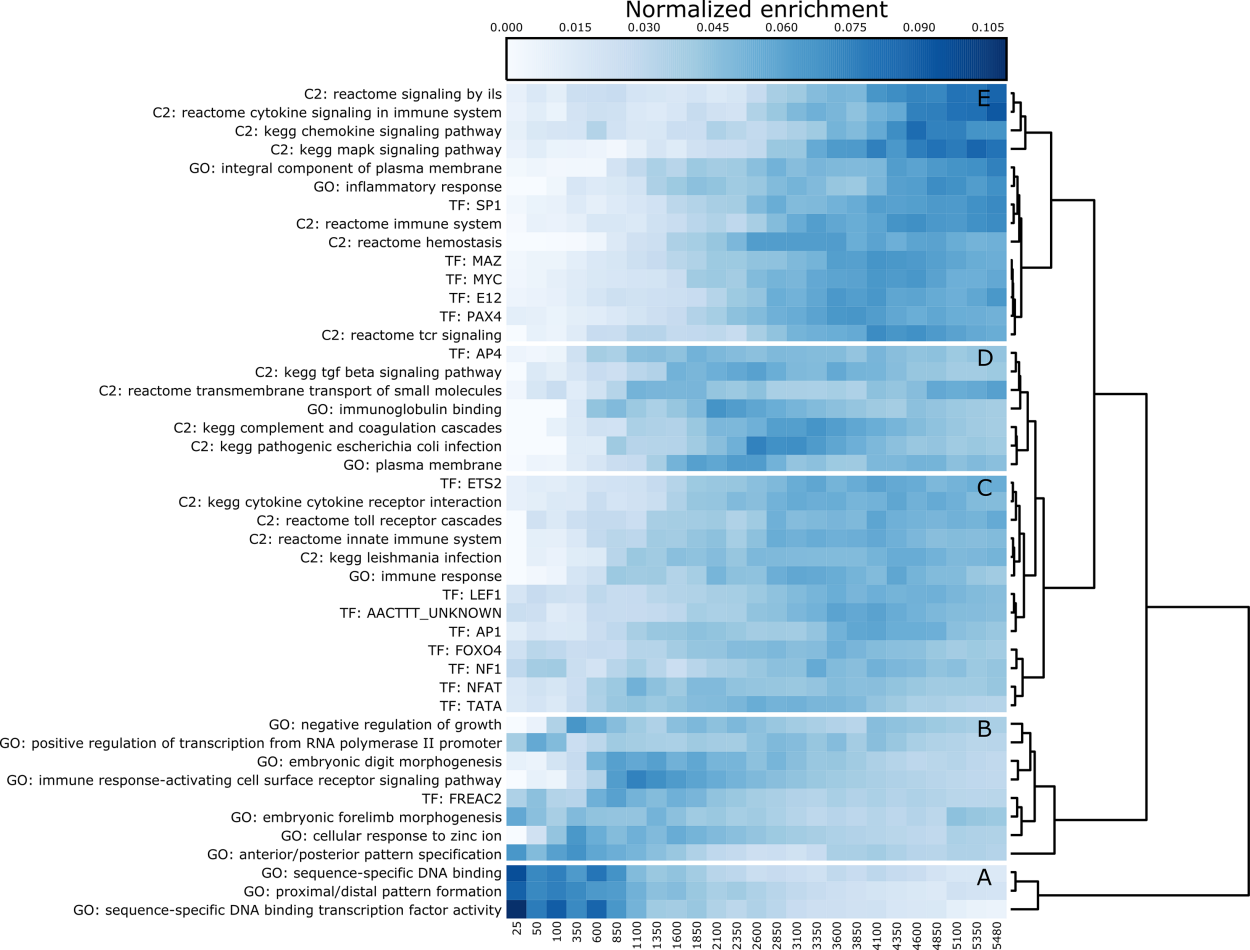
Clustergram of Top Enriched Pathway, TF, and GO terms. Concatenated enrichment profiles for GO, C2, and TF gene set collections, similar to Fig 3, ordered by hierarchical clustering of Euclidean distance between rows. Rows have been normalized by dividing by the row sum for visualization, intensity is proportional to normalized enrichment. Heatmap is partitioned into groups A-E based on hierarchical clustering. Clusters A, B, and C are primarily involved in the immune response and are enriched in gene subsets that include more genes. Clusters D and E are predominantly related to developmental and transcriptional regulation processes.

The top fifteen most enriched gene set profiles from each of GO, Canonical Pathways, and Transcription Factors were concatenated and hierarchically clustered to identify which gene sets are enriched in similar DE genes, as shown in Fig 4. The clustering identifies five groups of genesets that correspond primarily to either immune response or developmental functions (A-C, and D-E respectively in Fig 4). Transcription Factor genesets are clustered with pathway and GO genesets, indicating which co-regulated genes are associated with which biological functions. Further remarks on this result are found in the Discussion section.

### Association of Gene Expression with Clinical Covariates

Genes whose expression is associated with CAG-adjusted age at onset are potential genetic factors that modify the presentation of disease independent of CAG repeat length, though in the presence of the mutation, and thus may be useful as a biomarker in identifying patients at risk of early onset. Therefore, to identify genetic factors that may modify clinical covariates, each of the 28,087 confidently expressed genes was analyzed for association with CAG repeat length, CAG-adjusted residual age at onset, and scores representing cortical and striatal involvement using the Hadzi-Vonsattel (H-V) method[11]. Due to the significant association between age at onset and CAG repeat length, a CAG-adjusted residual age at onset variable was constructed with the model from Djousse et al[30] and used to test for association (see Methods).

Association was assessed using a linear regression model predicting normalized, normally-transformed counts (see Methods) from each covariate separately, adjusting for RNA integrity number RIN. No gene associations reached genome-wide significance after multiple hypothesis adjustment, though many reached nominal significance as described in Table 7 and Tables B, C, D, and E in S1 File. We did not find any significant association between gene expression in HD brains and either the striatal or cortical H-V involvement scores. While this may be a consequence of the relatively small sample size of twenty HD brains studied here, it is also worth noting that these brains exhibited a wide range of cortical (from 0.401 to 2.361) and striatal (from 2.132 to 3.820) involvement on the H-V scale. To identify potential confounding in the DE gene list by cortical involvement, we analyzed the DE gene counts to identify any with significant association with H-V cortical score (see Methods). None of the DE genes attained significance after multiple hypothesis adjustment, indicating the DE gene results are not confounded by cortical involvement.

**Table 7:** Protein coding genes associated with clinical covariates. P-values are nominal.

CAG Repeat Length			CAG Adjusted Onset			Cortical involvement score		
Gene	beta	p-value	Gene	beta	p-value	Gene	beta	p-value
C2CD3	-0.07136	0.000139	CAPN8	0.589035	0.000129	STRADB	0.271437	7.87E-05
NPBWR1	0.224468	0.00021	ARSF	-0.58752	0.000461	ABCF3	-0.36875	0.00038
GPR142	-0.1366	0.000275	BICD2	-0.22363	0.000474	BARD1	0.888169	0.000423
CEP95	-0.0913	0.000423	MYB	-0.68965	0.000766	TMEM190	0.582559	0.000514
C18orf42	0.207978	0.000583	GDF5	0.68	0.00121	GLUD1	0.621515	0.000522
NNAT	0.176257	0.000658	KLHL40	0.537238	0.001479	F2R	1.010939	0.00054
OFD1	-0.10494	0.000669	PODNL1	0.555785	0.001579	FAM64A	-1.01782	0.000547
SOX1	0.112901	0.000683	CRELD2	0.307269	0.001749	SDC4	1.069862	0.000552
PCDH8	0.232301	0.000734	PLEK2	-0.63464	0.001817	RIN2	0.827077	0.000677
NAA20	0.062964	0.000743	ZNF398	-0.25083	0.001828	ANGPTL4	1.498085	0.000752
SH3TC2	-0.24499	0.000823	EPS8L2	0.382135	0.002523	STOX1	0.703621	0.000783
RWDD2B	0.104283	0.000829	PAX5	-0.64915	0.002563	DLK2	-0.7786	0.000898
IGF1	0.199257	0.000846	GATSL1	-0.42252	0.002896	WWOX	0.440922	0.000991
PAPL	-0.21977	0.000869	ICMT	-0.24777	0.003183	RFC5	-0.32834	0.001024
DST	-0.13745	0.000877	NPY2R	-0.78898	0.003207	DPH2	-0.3034	0.001124
C1orf131	-0.06809	0.000889	POLA2	0.331568	0.003421	ETNPPL	0.980477	0.001187
GDNF	-0.15728	0.000909	PRPSAP1	0.245984	0.003581	PON2	0.718243	0.001353
PDCD2	0.034442	0.000965	TTC16	0.456621	0.003612	ELP4	0.602198	0.001368
NCKAP5	-0.14426	0.001001	C3orf52	-0.56693	0.003654	MYADM	-0.40523	0.001438
FAM194A	0.164749	0.001009	FAM127C	0.195028	0.004014	NR5A1	-0.65309	0.001475

## Discussion

We conducted mRNA transcriptional analyses in HD and control brains to identify altered gene expression profiles in this disease. To our knowledge, these are the first reported results from a gene expression analysis of high-throughput mRNA sequencing from post-mortem human HD and control brains. Widespread DE genes strongly implicate immune response, transcriptional dysregulation, and extensive developmental processes across all primary brain cell types (i.e. astrocytes, oligodendrocytes, microglia, and neurons). The genes from the DES-ranked list in Table 4 reveal a variety of disease related processes, implicating genetic signatures for different brain cell types as well as genes heavily associated with brain injury and neurodegeneration. The top two DES-ranked genes, *MBP* (myelin basic protein) and *GFAP* (glial fibrillary acidic protein), are typical markers used to identify oligodendrocytes and reactive astrocytes, respectively[31]. These proteins have also been implicated in immune processes, blood-brain barrier permeability, and response to injury in the central nervous system[31–33]. The next highest DES-ranked gene, *CLU* (clusterin), is associated with clearance of cellular debris, lipid recycling, apoptosis, and, as a stress-induced secreted chaperone protein, has been genetically associated with late-onset Alzheimer’s disease[34]. GLUL (glutamate-ammonia ligase) is a glutamine synthetase found primarily in astrocytes in the brain and is involved in neuron protection from excitotoxicity through the conversion of ammonia and glutamate to glutamine[35]. Alteration in *TUBB4A* (tubulin beta-4A chain), a major component of microtubules, has been associated with neurodegenerative diseases caused by hypomyelination with atrophy of the basal ganglia and cerebellum[36]. *AQP4* (aquaporin) is a specific marker for astrocytic endfeet and has been linked to Ca2+ induced edema[37]. *ENO2* (ennolase), a neuron-lineage-specific gene ranked 19^th^ by DES, has been identified as a marker for ischemic brain injury[38]. Although it is not included in the top list, the analysis also identified *CD40*, a protein uniquely expressed in activated microglia for antigen presentation in the brain[39]. Together, these genes suggest a systemic response in all brain cell types to stress and brain injury.

While some of the differences in gene expression that are observed in our studies are almost certainly a consequence of alterations in the cellular distribution in HD due to the loss of neuronal cells and the reactive response to degeneration in the HD brain, it is important to note that we did not find that the levels of gene expression in HD brains were related to the extent of cortical involvement. Specifically, while the HD samples in this study range from very low (H-V cortical score 0.401) to very high (H-V cortical score 2.361) levels of cortical involvement, levels of differentially expressed genes were not found to be significantly associated with H-V cortical score. Because the H-V cortical score comprehensively characterizes the level of involvement and cellular architecture of the HD brains studied, these findings suggest that the differentially expressed genes are not simply a reflection of altered distribution of cell types in the samples studied.

DAVID functional clustering analysis identified a number of functionally related clusters with overlapping genes. The network in Fig 2 illustrates that the immune system and developmental clusters are highly interrelated in their underlying genes, suggesting a link between these cellular processes. The detailed analysis of different gene subsets for enrichment of GO, Canonical Pathways, and Transcription Factors affords some insight into this relationship as illustrated in Fig 4. The top fifteen most enriched gene set profiles from each collection were concatenated and hierarchically clustered to identify which gene sets are enriched in similar DE genes. The clustering identifies five distinct clusters that are functionally organized into coherent groups (labeled A-E in Fig 4). Clusters A, B, and C are primarily involved in the immune response and are enriched in gene subsets that include more genes. Transcription factors *SP1*, *MAZ*, *MYC*, *E12*, and *PAX4* are enriched in similar sets of DE genes that are also involved in inflammatory and immune response, suggesting these functions are transcriptionally related. Clusters D and E are predominantly related to developmental and transcriptional regulation processes, and are clustered with transcription factor *FREAC2* (Forkhead Box F2, also known as *FOXF2*) which, as a member of the forkhead family of transcription factors, is potentially implicated in development, organogenesis, regulation of metabolism, and immune system processes[40].

The strong implication of immune response and neuroinflammation in this study is consistent with prior reports as a critical aspect of the human response to HD[41–43]. The set of DE genes is highly enriched for multiple immune system processes, including both innate and adaptive immune response, implicating a tissue-wide immune response at multiple cellular levels. The presence of the proinflammatory genes *NFkB* and interleukins (*IL8*, *IL9*, *IL15*, *IL18*) is strong indication of an innate immune response and is previously reported in the HD literature[41–43].

Except for our recent miRNA finding[10], the Hox locus has not previously been implicated in HD in model or human systems. The extent of altered developmental genes is quite striking and affords no immediate interpretation since the enriched developmental processes seem to be specific to cell types that have no obvious role in the central nervous system (i.e. skeletal, limb morphogenesis, etc.). This apparently non-specific developmental enrichment might therefore be a consequence of profound transcriptional changes related to the extreme inflammatory stress experienced by the affected brain regions as well as transcriptional dysregulation due aberrant interactions between TFs and mutant *HTT* protein fragments. It is still unclear whether a subset or if all brain cell types are responsible for this signal, and elucidation of the source of the developmental gene transcription may provide further insight into the cell type specificity of transcriptional dysregulation.

This dataset suggests the calpain family of proteolytic proteins plays a role in HD. Calpains have a direct role in the cleavage of mutant Htt into toxic fragments[44] and the inhibition of these proteins leads to decreased neuronal toxicity in *in vitro* settings[45]. Three calpains, *CAPN2*, *CAPN7*, and *CAPN11*, are significantly DE in this dataset, where 2 and 7 are highly abundant and up-regulated in HD while 11 shows low expression and is down regulated. Calpains are typically activated by elevated intracellular Ca+2 levels[46] and there is significant evidence in this dataset that genes responsive to calcium and other ionic metals are activated. Four of the eight calmodulin related genes (*CALM1*, *CALM2*, *CALML3*, *CALML4*) are DE in the dataset, and are all significantly down regulated with the exception of *CALML4* (LFC -0.55, -0.35, -0.97, 0.42, respectively). Calcium plays a key role in apoptotic phagocytosis and the inflammatory response[47,48], processes that are strongly implicated in this dataset, and disrupted calcium concentration has been implicated in HD and neurodegeneration in general[49,50]. Among the enriched GO categories are calcium-dependent protein binding, calcium-dependent phospholipid binding, cellular response to cadmium ion, and cellular response to zinc ion. Metallothioneins appear as one of the most enriched DAVID functional clustering results, with nearly every metallothionein 1 subtype DE in the dataset (all except MT1B). Altogether, this dataset strongly implicates the presence of metal ion disequilibrium in the HD context. Though the presence of ion disequilibrium is strongly implicated by this study, it is unclear whether this effect is a cause or a consequence of the toxic effects of mutant *Htt*.

A popular hypothesis asserts that mitochondrial dysfunction contributes to neurodegeneration in HD[51–53]. Dysregulation of mitochondrial function in HD is thought to be induced by disrupted cytoplasmic Ca2+ concentrations[51] which lead to alterations in bioenergetic processes and mitochondrial morphology[52]. Several of the signals observed in this study suggest an imbalance in calcium ion homeostasis in the human HD brain as described above, which supports the hypothesis that mitochondrial dysfunction is implicated in human HD. However, none of the mitochondrial genes are DE in this dataset.

In contrast to this study, Hodges et al[54] found no detectable gene expression changes for HD in post mortem BA9 tissue. Nonetheless, there are consistencies between our findings. First, although overall gene expression was observed to be down regulated in the striatum for Hodges et al, the distribution of fold changes for BA9 in both studies indicate overall up regulation. Second, and more significantly, there is suggestive overlap of enriched biological processes between the two datasets across brain regions. Specifically, they observed that central nervous system and neuronal developmental genes, ion transport, microtubule, and vesicle-related processes were enriched, signals also observed in this study.

The discovery of thousands of statistically significant differences in gene expression presented a major challenge to the interpretation of this dataset. The DAVID analysis, which is specifically designed to interpret large gene lists, was not sufficiently detailed to readily provide insight about which genes were involved in which functions, nor did the tool organize its output in a way that presents how different enriched genesets are related. The method developed here addresses both of these issues, and allows the use of different statistical enrichment methods, as appropriate, for different gene sets. It also combines and visualizes the enrichment information in such a way as to facilitate generating specific hypotheses concerning which genes are related through their enrichment profiles. The link between genes that are regulated by TFs known to interact with mHtt fragments and their immunological functions (Fig 4 cluster A) proposes a mechanism by which mHtt may play a toxic role to cells, namely via transcriptionally altering genes involved in the immune response. FOXF2 was also identified as a TF that is potentially responsible for aspects of both the inflammatory and developmental gene expression changes (Fig 4 cluster D). These insights were not obvious from the DAVID results, demonstrating the utility of our novel analytical methodology.

These data represent the most comprehensive characterization of genome-wide gene expression in human HD subjects to date. The broad scope of changes across biological functions and cell types establishes HD as a systemic disease of the brain, implicating not only neurons but also the primary glial cell types. This new molecular evidence supports previous imaging-based observations of cortical and whole-brain structural changes in HD[55–57]. The immune response is intrinsically intercellular in its activation and function, cued by the complex interaction of stressed neurons and the reactive glial cells of the central nervous system immune response. This brings into focus the importance of considering the HD brain as a whole organ, and important advances in understanding and mitigating HD pathogenesis may be gained by developing and studying models of these complex multi-cellular interactions. In particular, *in vitro* studies of human-derived neuronal HD cell line models and HD mouse models cannot capture the complexity of the human brain microenvironment, an especially important point for mouse models due to the compelling differences between the human and murine inflammatory response[58]. It remains to be shown precisely which cell types are responsible for which aspects of the biological response observed in this study. Similarly, it is not known how the immune and developmental DE genes are related, and whether some complex combination of these genes can be shown to modulate clinical features of disease, in particular age of onset. It is conceivable that subjects with a different or more extreme immune response may experience neurodegeneration differently than others, and we hypothesize that this avenue of research will yield important advances in our understanding of HD pathogenesis.

## Methods

### Sample Information

Frozen brain tissue from prefrontal cortex Brodmann Area 9 (BA9) was obtained from the Harvard Brain and Tissue Resource Center McLean Hospital, Belmont MA, the Human Brain and Spinal Fluid Resource Center VA West Los Angeles Healthcare Center (Los Angeles, California) and Banner Sun Health Research Institute[59] (Sun City, Arizona). Twenty Huntington's disease (HD) samples and forty nine neurologically normal control samples were selected for the study (See Tables 1 and 2). Age at death and RIN were significantly different between cases and controls (p = 0.01 and p = 0.006, respectively, by Welch two sample t-test). The HD subjects had no evidence of Alzheimer or Parkinson disease comorbidity based on neuropathology reports. All samples were male. Neuropathological information for the HD samples includes the Vonsattel grading[4], as well as striatal and cortical scoring recently described by Hadzi et al.[11]. Additionally, CAG repeat size and age at onset were known for the HD samples (Table 1).

### Human Subjects

This study has been designated exempt (Protocol # H-28974) by the Boston University School of Medicine Institutional Review Board, as no human subjects were studied and all data are derived from post-mortem human brain specimens.

### mRNA Sample Preparation and Sequencing

For each brain sample, grey matter from the cortical ribbon was dissected by hand with a target mass of 0.08 g and used for RNA extraction. 1 ug of RNA was used to construct sequencing libraries using Illumina’s TruSeq RNA Sample Prep Kit according to the manufacturer’s protocol. All sample dissections and RNA extractions were performed by the same individual. RNA Integrity Number (RIN) was measured by the Agilent Bioanalyzer to assess RNA quality prior to sequencing. In brief, mRNA molecules were polyA selected, chemically fragmented, randomly primed with hexamers, synthesized into cDNA, 3’ end-repaired and adenylated, sequencing adapter ligated and PCR amplified. Each adapter-ligated library contained one of twelve TruSeq molecular barcodes. Multiplexed samples were equimolarly pooled into sets of three samples per flowcell lane and sequenced using 2x101bp paired-end runs on Illumina’s HiSeq 2000 system at Tufts University sequencing core facility (http://tucf-genomics.tufts.edu/). Demultiplexing and FASTQ file generation (raw sequence read plus quality information in Phred format) were accomplished using Illumina’s Consensus Assessment of Sequence and Variation (CASAVA) pipeline. Sequences were aligned against the hg19 reference genome[60] using tophat v2.0.6[61], with non-default parameters (see S1 Text).

### Gene Expression Quantification, Data Cleaning, and DE Analysis

Aligned reads were mapped to the Gencode v17 annotation[12] using the htseq-count tool in the HTSeq v0.5.3p9 package[62] with the intersection non-empty strategy. Genes that had less than half of HD and control samples with nonzero counts were filtered from the analysis due to low signal. No samples were identified as outliers, and extreme gene measurements considered outliers were adjusted as described in S1 Text. Outlier-trimmed raw counts were used in subsequent analyses. DESeq2[63] was used to identify DE genes between HD and control, adjusting for age at death binned into intervals 0–45, 46–60, 61–75, and 90+ and a categorical RNA Integrity Number (RIN) variable indicating RIN>7 as covariates. Genes with FDR<0.05 were considered DE.

### DAVID, GO, and MsigDB Enrichment Calculation

The DAVID[13,14] functional enrichment clustering tool set to the lowest clustering stringency was used on the top 3000 DE genes to identify groups of enriched functions. DAVID limits the number of genes submitted for analysis to 3000. Clusters were considered significant if the cluster score was greater than–log10(0.05). Separate enrichment analyses were performed using the Gene Ontology (GO) annotation database[15], the MsigDB[16] C2 Canonical Pathways gene sets, and the MsigDB C3 Transcription Factor target gene sets. Enrichment was calculated for subsets of top DE genes separately, i.e. enrichment analysis was performed on the top 25 genes, then on the top 50, and so on. GO term enrichment was performed using topGO[17] with the “weight01” algorithm and “fisher” statistic, and custom scripts in the R statistical environment[64]. Enrichment of MsigDB Canonical Pathways and Transcription Factor genesets was performed with custom R scripts using the “fisher.test” and “p.adjust” routines. Once enrichment profiles for each geneset was computed, the genesets were ranked based on the most significant enrichment found in any gene group. The top 15 most significant geneset enrichment profiles from each database were selected and concatenated into a single enrichment matrix with genesets as rows and gene groups as columns. The rows of this matrix were clustered using agglomerative hierarchical clustering with Ward linkage. Further processing of enrichment results was performed using custom scripts to generate plots in python with matplotlib[65], ipython notebook[66], and pandas[67].

### Association with Clinical Covariates

DESeq2 normalized counts were transformed using the Variance Stabilizing Transform (VST) available in the same package to produce approximately normally-distributed gene expression values. After the normal transformation, the standard linear regression model becomes appropriate for evaluating association with covariates. Linear models predicting VST transformed counts from each clinical covariate after adjusting for RIN were run for each gene in the R statistical environment. P-values were adjusted using the “p.adjust” function in R using the FDR method. To assess which DE genes were associated with H-V cortical score, DESeq2 was used to model read counts as predicted by H-V cortical score adjusting for RIN for each gene, adjusted for multiple hypothesis with the “p.adjust” function in R using the FDR method.

### Replication of DE Genes by RT-qPCR in an Independent Sample Set

An independent set of 33 HD and 31 control prefrontal cortex brain samples not used in the sequencing study were subjected to RT-qPCR to replicate the findings of this study. RNA was reverse transcribed using iScript cDNA Synthesis Kit (Bio-Rad). Reverse transcriptase quantitative polymerase chain reaction (RT-qPCR) was carried out for all genes of interest in each sample using TaqMan Gene Expression Assays (Life Technologies) on an ABI 7900HT Real-Time PCR system, according to the manufacturer’s protocol. All probes were human and covered all transcripts: HOXC10 (Assay ID Hs00213579_m1) and NFKBIA (Assay ID Hs00355671_g1) probes were used. Peptidylprolyl isomerase A (PPIA, catalog #4333763F) and beta glucuronidase (GUSB, catalog # 4333767F) were used as endogenous controls. Samples were run in triplicate at 200ng mRNA per reaction. For HOXC10, presence or absence of transcripts was assessed by whether a critical threshold (CT) value was determined or undetermined, respectively, at the threshold chosen by Applied Biosystems SDS software v2.4. For NFKBIA, wells that caused the variance of the corresponding set of replicates to exceed 0.2 were marked as outliers and excluded from the analysis (9 such replicates from unique sample/assay combinations were excluded). To normalize sample input, deltaCT values were calculated for each sample by subtracting the average CT for a target gene by the averaged CT for both control genes. Two sample t-tests assuming equal variance with deltaCT values were used for statistical analysis.

### Validation of DE Genes by RT-qPCR

The RNA used in the RT-qPCR was from the same extraction as submitted for sequencing and thus was intended to be a technical validation of the sequencing results. Validation samples were prepared and processed for RT-qPCR in the same manner as the replication samples, described above. All probes were human and covered all transcripts: AHNAK nucleoprotein (AHNAK, Assay ID Hs01102463_m1), paired-like homeodomain (PITX, Assay ID Hs00267528_m1), aquaporin 4 (AQP4, Assay ID Hs00242342_m1), solute carrier family 38, member 2 (SLC38A7C, Assay ID Hs01089954_m1), gap junction protein, alpha 1, 43kDa, (GJA1, Assay ID Hs00748445_s1), and tumor protein p53 inducible nuclear protein 2 (TP53INP2, Assay ID Hs00894008_g1) probes were used. As with the replication study, PPIA and GUSB were used as endogenous controls. Samples were run in triplicate at 30ng per reaction. Wells with critical threshold (CT) values higher than 3 standard deviations were removed from analysis. To normalize sample input, deltaCT values were calculated for each sample by subtracting the average CT for a target gene by the averaged CT for both control genes. Wells that were undeterminable were replaced with the maximum number of cycles (40) in order to calculate deltaCT. Two sample t-tests assuming equal variance with deltaCT values were used for statistical analysis.

## Supporting Information

S1 File Table A: DESeq2 DE Statistics on all genes. Table B: Genes associated with CAG adjusted age of onset. Table C: Genes associated with CAG size. Table D: Genes associated with cortical score. Table E: Genes associated with striatal score. Table F: DE Hox genes. (XLSX)

S1 Text Extreme count outlier trimming strategy, tophat alignment parameters. (PDF)
