# Correction: RNA Sequence Analysis of Human Huntington Disease Brain Reveals an Extensive Increase in Inflammatory and Developmental Gene Expression

**DOI:** 10.1371/journal.pone.0160295

**Published:** 2016-07-25

**Authors:** Adam Labadorf, Andrew G. Hoss, Valentina Lagomarsino, Jeanne C. Latourelle, Tiffany C. Hadzi, Joli Bregu, Marcy E. MacDonald, James F. Gusella, Jiang-Fan Chen, Schahram Akbarian, Zhiping Weng, Richard H. Myers

Table 1 and Table 2 appear incorrectly in the published article. Please see the correct Tables 1 and 2 and their captions here.

**Table 1:** HD sample statistics.

Sample ID	PMI	Age of Death	RIN	mRNA-Seq reads	Age of Onset	Duration	CAG	Vonsattel Grade	H-V Striatal Score	H-V Cortical Score
H_0001	37.25	55	7.1	74,635,390	44	11	45	3	2.661	0.922
H_0002	5.75	69	7.5	71,015,288	63	6	41	3	2.644	1.081
H_0003	20.5	71	7.0	77,385,918	52	19	43	3	2.428	1.707
H_0005	19.15	48	6.9	82,366,794	25	23	48	4	3.820	1.939
H_0006	NA	40	6.2	77,123,676	34	6	51	4	3.522	1.431
H_0007	8	72	8.5	63,294,390	55	17	41	3	2.593	0.849
H_0008	21.3	43	7.4	71,056,116	28	15	49	3	2.701	1.701
H_0009	3.73	68	7.8	66,169,262	45	23	42	3	2.668	1.701
H_0010	6.16	59	8.3	65,341,820	35	24	46	3	2.621	1.200
H_0012	12.75	68	6.0	83,110,358	52	16	42	3	2.661	1.077
H_0013	25.1	57	6.1	71,320,688	40	17	49	3	2.911	1.491
H_0539	14.5	54	6.5	124,222,130	42	12	45	3	2.132	0.401
H_0657	24.3	61	8.1	136,764,622	36	25	45	4	3.290	1.604
H_0658	11	48	7.8	85,591,704	42	6	44	3	2.410	0.978
H_0681	19.06	69	7.0	78,493,314	50	19	42	3	2.484	1.088
H_0695	16.15	55	7.9	86,412,654	36	19	45	4	3.581	2.062
H_0700	15.66	50	8.0	78,329,378	33	17	47	3	2.741	1.202
H_0726	14.75	50	9.2	86,025,890	27	23	48	4	3.598	1.201
H_0740	13.58	75	6.4	101,997,434	60	15	42	3	2.621	2.361
H_0750	16.16	53	6.0	122,909,122	38	15	48	4	3.260	1.010

**Table 2:** Control sample statistics.

Sample ID	PMI	Age of Death	RIN	mRNA-Seq reads
C_0012	19	66	7.1	118,327,116
C_0013	15	69	7.8	89,478,160
C_0014	21	79	8.0	65,377,604
C_0015	10	61	8.2	123,746,070
C_0016	20	58	8.4	67,758,208
C_0017	21	70	8.2	72,238,818
C_0018	17	66	8.5	64,688,322
C_0019	19	73	7.5	108,683,922
C_0020	24	60	7.9	83,696,384
C_0021	26	76	7.3	79,487,172
C_0022	17	61	7.8	73,133,936
C_0023	18	62	6.6	94,493,436
C_0024	26	69	8.7	62,989,822
C_0025	25	61	8.1	55,810,684
C_0026	11	88	7.1	72,581,752
C_0029	13	93	6.4	59,386,108
C_0031	24	53	7.3	73,283,170
C_0032	24	57	8.3	70,994,352
C_0033	15	43	7.5	69,505,712
C_0035	21	46	7.6	62,300,754
C_0036	17	40	7.5	63,961,372
C_0037	28	44	8.3	60,288,132
C_0038	20	57	7.7	61,019,098
C_0039	15	80	7.3	74,892,650
C_0050	2	74	8.5	85,310,070
C_0053	2	69	8.4	167,044,880
C_0060	2	76	7.5	103,952,680
C_0061	3	78	7.6	95,393,100
C_0062	2	87	8.7	83,773,400
C_0065	2	86	8.7	115,714,502
C_0069	24	54	8.3	128,459,102
C_0070	19	68	6.3	145,087,692
C_0071	21	106	7.6	86,840,836
C_0075	23	52	7.4	99,946,984
C_0076	30	46	8.2	85,890,116
C_0077	21	36	8.5	80,103,722
C_0081	26	55	7.6	82,917,984
C_0082	18	57	7.8	123,118,398
C_0083	32	66	8.4	80,696,360
C_0087	19	64	8.7	77,198,978
C_0002	2	73	7.7	120,108,434
C_0003	2	91	7.9	38,420,004
C_0004	2	82	8.6	75,850,406
C_0005	2	97	9.1	150,661,916
C_0006	5	86	8.6	63,607,838
C_0008	2	91	8.7	66,131,458
C_0009	3	81	6.0	69,284,092
C_0010	2	79	8.4	60,542,776
C_0011	2	63	6.5	93,702,684
