# Supplementary material for: RNA Sequence Analysis of Human Huntington Disease Brain Reveals an Extensive Increase in Inflammatory and Developmental Gene Expression
Source: PLoS One. 2015 Dec 4;10(12):e0143563. doi: 10.1371/journal.pone.0143563 (PMC4670106; doi:10.1371/journal.pone.0143563)
Supplement: S1 Text — (PDF) [file pone.0143563.s002.pdf]

Extreme count outlier trimming strategy

It has been documented that DESeq2 is sensitive to outliers and performs poorly when a gene is expressed in one condition and absent in another[1,2]. The package attempts to overcome these limitations by flagging and removing or trimming outlier samples using a statistical threshold related to Cook's distance. We found that this strategy performed poorly on some of our most highly differentially expressed genes due to noise or condition-exclusive expression in our samples. In particular, the most differentially expressed gene in our analysis, PITX1, was found to be insignificant when analyzed by DESeq2 with certain outlier trimming thresholds. After examining the genes flagged as outliers by DESeq2, we devised a new strategy for adjusting outlier counts based on the count contribution of each sample as follows. For each gene with DESeq2 normalized counts:

1. Divide each sample count by the sum of counts (i.e. sample count proportions)
2. Identify samples that have >*p* sample count proportion
    a. If no samples are identified, return the most recent set of adjusted counts
    b. Else, shrink the identified samples toward the largest sample for which P(x)<p:
    $\hat{x_i} = x_{max<p} + \lambda(x_i - x_{max<p})$
3. Go to 1, repeat until no samples exceed *p* count proportion

This strategy assumes that samples with disproportionate count contribution are outliers and that the order of samples is correct and the magnitude is sometimes not. The order of the samples is thus always maintained, and the shrinking does not introduce new false positives beyond what would already be in the dataset. The maximum proportion of reads allowed in one sample, *p*, and the shrinkage factor lambda were both set to 0.2. With these parameters, 685 samples in 646 genes were trimmed, removing a total of approximately 870,290 normalized counts.

Tophat alignment parameters

The parameters used in the tophat alignment were: --read-mismatches=3 --read-edit-dist=3 --max-multihits=20 --splice-mismatches=1 --microexon-search --coverage-search --mate-inner-dist=50 --mate-std-dev=50.

References:

1. Soneson, C. & Delorenzi, M. A comparison of methods for differential expression analysis of RNA-seq data. *BMC Bioinformatics* **14,** 91 (2013).

2. Rapaport, F. *et al.* Comprehensive evaluation of differential gene expression analysis methods for RNA-seq data. *Genome Biol.* **14,** R95 (2013).
